# Current advances and future prospects of cell reprogramming in progeroid syndromes

**DOI:** 10.3389/fcell.2025.1546423

**Published:** 2025-02-19

**Authors:** Lucas Moledo-Nodar, Víctor Celemín-Capaldi, Alejandro P. Ugalde, José M. P. Freije

**Affiliations:** ^1^ Departamento de Bioquímica y Biología Molecular, Facultad de Medicina, Instituto Universitario de Oncología, Universidad de Oviedo, Oviedo, Spain; ^2^ Instituto de Investigación Sanitaria del Principado de Asturias (ISPA), Oviedo, Spain; ^3^ Centro de Investigación Biomédica en Red de Cáncer (CIBERONC), Madrid, Spain

**Keywords:** cell reprogramming, progeria, stem cell, IPSC, aging

## Abstract

Cell reprogramming consists in the reverse process to cell differentiation, making cells lose their identity and age-related characteristics and granting an increased potential for proliferation and redifferentiation on different lineages. This process holds immense potential for the treatment of several pathologies, including progeroid syndromes, diseases that recapitulate the symptoms seen in physiological aging in an accelerated manner. Among the recent advances on the use of cell reprogramming in the context of progeroid syndromes, the interventions based on partial reprogramming, consisting on the dedifferentiation of cells only up to a point in which they lose age related characteristics but keep their identity, stand out. This partial reprogramming can be achieved both using the forced expression of transcription factors or cocktails of small molecules that regulate different biological processes. While all these advances are promising, the use of cell reprogramming in the treatment of progeroid syndromes still faces several challenges, such as the development of methods that allow for an efficient delivery of cell reprogramming factors *in vivo* and fine tuning of the dose used. Furthermore, these approaches should be accompanied by treatments targeting the original cause of the disease or they could be proven futile in the long term.

## 1 Introduction

Cell reprogramming refers to the dedifferentiation of adult somatic cells to produce pluripotent embryonic cells (ES). Historically, cell differentiation was considered a unidirectional and irreversible process. In this context, the biologist Conrad Waddington referred to the “epigenetic landscape” metaphor to describe the cell differentiation process. According to this metaphor, embryonic cells can be compared to balls moving through a landscape of valleys and plateaus that represents the process. As the cells descend to lower heights, they progressively loose differentiation potential until they reach the bottom of the valley, which corresponds to the acquisition of a definitive cell type (Pesaresi et al., 2019). However, in the 1960s, John Gurdon, using *Xenopus laevis* models, demonstrated that cell fate could be reversed by somatic cell nuclei transfer (SCNT) to denucleated eggs, with the consequent formation of viable zygotes (Gurdon, 1962). Later, the same technique was performed in several mammals, such as sheep and mice (Wilmut et al., 1997; Wakayama et al., 1998). Furthermore, by fusing ES cells and human somatic cells, hybrid cells were obtained with the ability to form the three germ layers in embryonic bodies *in vitro*, through the activation of embryonic genes and the silencing of somatic genes in the hybrid chromosomes (Tada et al., 2001; Cowan et al., 2005).

**FIGURE 1:**
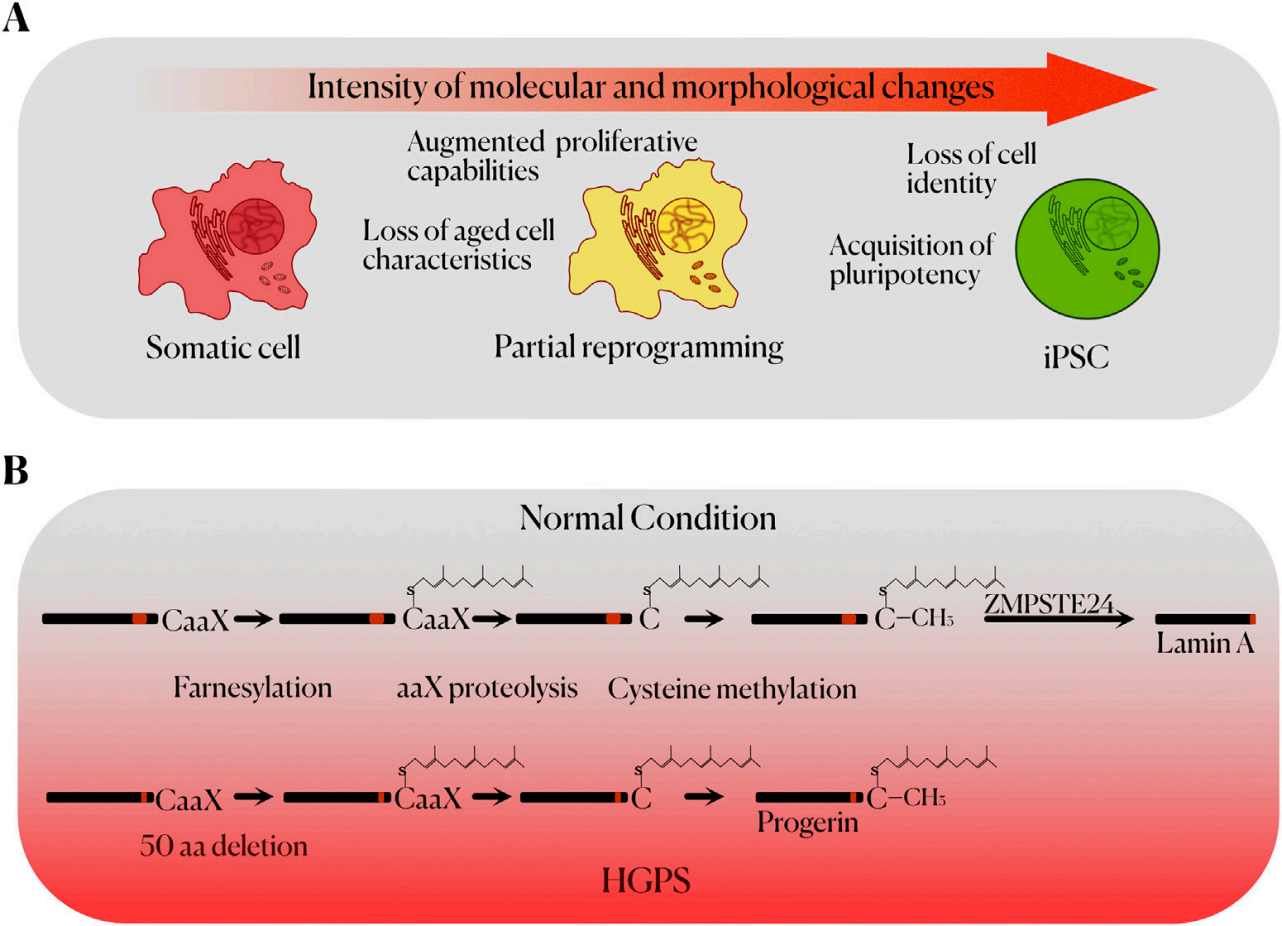
**(A)** Stages of cell reprogramming up to the acquisition of pluripotency, including the main characteristics of each state. **(B)** Molecular mechanism of Hutchinson-Gilford Progeria Syndrome. The normal post-translational processing of prelamin A (left) to mature lamin A (right) is represented on top. The defective processing of the mutant form of prelamin A (progerin) seen in HGPS, due to the lack of 50 aa in progerin, prevents the final proteolytic processing of the protein, leading to its accumulation and causing genomic instability and cellular toxicity.

The cellular reprogramming achieved using these techniques indicated the existence of factors in the cytoplasm of oocytes and ES cells that play an important role in conferring pluripotency. A major advance in this field was made when Yamanaka and Takahashi, through retroviral transduction of the transcription factors OCT4, SOX2, KLF4 and C-MYC in mouse and human embryonic and adult cells, were able to generate induced pluripotent stem cells (iPSCs). In addition to their differentiation potential, these cells showed similarities with ES cells in their morphology, proliferation rate, gene expression, surface markers, telomerase activity and their ability to form teratomas (Figure 1A) (Takahashi and Yamanaka, 2006; Takahashi et al., 2007). OCT4 and SOX2 upregulate embryonic genes, while inhibiting the expression of genes associated with cellular differentiation. In this way, OCT4 is expressed in every cell of the early stages in murine and human development, upregulating the expression of genes related to pluripotency, self-renewal, and stem cell maintenance (Jerabek et al., 2014). SOX2 also plays an important role in early development, regulating the expression of pluripotency genes (Novak et al., 2020). KLF4 is expressed in several tissues and is characterized by its ability to be a transcriptional activator or suppressor depending on the context. One of KLF4 target genes is *NANOG*, which is critical for the maintenance of pluripotency (Chhabra, 2017). Finally, C-MYC can upregulate up to 15% of the genes present in the human genome through chromatin structure modification, being involved in numerous molecular pathways (Knoepfler et al., 2006). Experimental results, however, show that KLF4 and C-MYC are not as essential for the generation of iPSCs as OCT4 and SOX2 (Yu et al., 2007).

The production of iPSCs has allowed researchers to study tissues affected by different diseases, allowing the generation of cellular models that facilitate the search for specific treatments (Tang et al., 2016). These experimental approaches have been very useful in the research of progeroid syndromes, rare genetic disorders that recapitulate many of the alterations also present in physiological aging (Carrero et al., 2016). These syndromes are usually caused by mutations in the components of the nuclear envelope or in genes involved in DNA repair pathways.

Hutchinson-Gilford progeroid syndrome (HGPS) is one of the most studied premature aging disorders, despite only having a prevalence of one in 20 million people (Gordon et al., 2018). At birth, patients usually do not present any relevant symptoms or signs. However, at an early age they begin to acquire typical characteristics of aging, such as loss of hair, skin tightness, generalized lipodystrophy or osteoporosis (Gordon et al., 2014; Shin and Worman, 2022). HGPS patients usually have an average lifespan of 14.6 years with the most common cause of death being cardiovascular diseases (Olive et al., 2010).

HGPS originate from a *de novo* mutation in the *LMNA* gene, which encodes lamins A and C, nuclear envelope proteins with a high importance in the maintaining of the nuclear structure. The most frequent mutation that causes HGPS (c.1824C>T; p.G608G) activates a cryptic splicing site in exon 11 of *LMNA*, which results in an aberrant splicing and the production of a truncated protein called progerin. This truncated protein lacks 50 amino acids from the normal sequence of prelamin A (Carrero et al., 2016). Due to this, the protein does not undergo complete post-translational processing, thus being accumulated in the nuclear envelope while permanently farnesylated and methylated, acquiring toxic properties and causing morphological and functional alterations (Goldman et al., 2004) (Figure 1B). In this regard, the appearance of nuclear blebs and significant changes in the organization of the chromatin are common (Carrero et al., 2016). This is associated with a higher susceptibility to DNA damage, as well as a lower capacity for DNA repair (Camozzi et al., 2014). Accelerated telomere shortening is also observed in cellular models of HGPS (Kudlow et al., 2008). Furthermore, defects in the nuclear lamina are also related to abnormal mitosis, and a decrease in the proliferative capacity of stem cells (Carrero et al., 2016). DNA damage and telomere shortening favour cellular senescence, which in progeroid murine models is associated with increased levels of p53 (Varela et al., 2005). Alterations are also observed in many metabolic pathways, related to growth hormone (GH), insulin-like growth factor (IGF-1) and autophagy; as well as an increased production of proinflammatory cytokines (Carrero et al., 2016). Therefore, the generation of iPSCs from HGPS donors can be useful in establishing models that recapitulate the development of some aspects of these morphological and functional alterations, which can be interesting in the search for new biomarkers and possible therapeutic targets (Yu et al., 2007).

## 2 Current state of cell reprogramming approaches in progeria research

The use of stem cells in the treatment of several pathologies has already been widely discussed and tested (Tręda et al., 2024). While there are several possible sources to obtain stem cells, in this perspective we will limit ourselves to iPSCs. There are several scenarios in which iPSCs have been shown to be useful in the research about progeroid syndromes. The first use case would be as cellular models for *in vitro* research. Obtaining different cell types from donor patients can be challenging, especially in the case of ultra-rare conditions such as progeroid syndromes. In this sense, several different cell types, such as smooth muscle and endothelial cells, have been developed using cell reprogramming methods (Bersini et al., 2020). These cellular models represent extremely valuable tools in the research about progeroid syndromes, as they provide cell types previously unavailable to be cultured and studied. There are also, however, several advances *in vivo*.

Even though treatments based on iPSCs are promising, many challenges remain, as the use of stem cells *in vivo* has been found to be quite a complex matter. On the one hand, cell therapy has not shown consistent results for many diseases, such as stroke, heart and neurodegenerative diseases or diabetes (Cerneckis et al., 2024). In many cases cell therapy shows promising results but does not recover the full extent of the damage or works just for a limited amount of time, since the source of the disease is not corrected (Tręda et al., 2024). Another problem usually found while testing stem cell-based therapies is the potential tumorigenicity of the cells used. Both administered iPSCs and those produced via *in vivo* cell reprogramming have been found to generate teratomas in mice (Abad et al., 2013; Lee et al., 2013; Senís et al., 2018). This is evidently a challenge to overcome in the use of iPSCs for the treatment of human diseases. Despite these problems and challenges, stem cell-based therapies could show a particularly promising potential for the aforementioned progeroid syndromes (Ocampo et al., 2016a; Cipriano et al., 2024), since the exhaustion of stem cell populations is a hallmark shared by both most progeroid syndromes and physiological aging (Espada et al., 2008; López-Otín et al., 2023). In this context, the use of iPSCs could be an option to replenish these populations and potentially ameliorate the symptoms characteristic of progeroid syndromes. Since the administration of iPSCs to most tissues poses a great deal of challenges, the most viable option would be *in vivo* cell reprogramming. Several advances have been made in this regard, originally with the achievement of cell reprogramming *in vivo* (Abad et al., 2013) through the expression of the four Yamanaka factors. Further studies showed that cell reprogramming *in vivo* augments the regeneration capacity in the liver of mice. This however also caused a failure in liver function and the death of the treated mice (Senís et al., 2018; Hishida et al., 2022; Parras et al., 2023). This concept has also been explored in aged mouse models. In this case, transgenic mice carrying the four Yamanaka factors (OSKM) in an inducible expression polycistronic cassette were treated to achieve either repeated transitory pulses or a single stronger pulse of expression of this cassette (Ocampo et al., 2016b; Browder et al., 2022; Chondronasiou et al., 2022). This resulted in limited cell reprogramming, which does not involve cell identity loss, avoiding teratoma formation and most of the deleterious effects concomitant with *in vivo* cell reprogramming. While representing a huge step forward in the use of cell reprogramming *in vivo*, partial reprogramming is a delicate and complicated process. If the dose of the different reprogramming factors (OSKM or only OSK) is not carefully tuned either the appearance of teratoma, the failure of the affected organs due to loss of cellular identity or the lack of the different beneficial effects can occur.

Partial reprogramming has also been achieved in murine models of accelerated aging without the use of allogenic DNA insertion, via the expression of OSK through the use of Adeno Associated Viruses (AAVs) (Sahu et al., 2024) or the activation of endogenous *Oct4* with Au nanoparticles carrying a dCas9 activator system (Kim et al., 2023). In both cases mice showed an amelioration of the symptoms associated with HGPS and an extension of their lifespan, while the lifespan extension is yet to be proven in WT aged mice. In that case, both epigenetic and transcriptomic firms were used to evaluate the effectiveness of the rejuvenation protocol. This could be seen as problematic, as reprogramming factors directly act on the epigenetic and transcriptomic landscapes of the cells, and therefore it could be argued that these changes are only proof of the correct expression of the different reprogramming factors in the treated animals. However, these results are reinforced by more complete experiments done on mouse models of accelerated aging. Progeroid mouse models are valuable resources that phenocopy physiological aging and allow for much shorter timeframes for these studies, although the differences between pathological and normal aging cannot be ignored. Progeroid mice used in partial reprogramming experiments share the same transcriptomic and epigenetic changes observed in aged WT mice treated for partial reprogramming, while yielding more conclusive experimental results. Partial reprogramming results also seem to vary between tissues depending on the protocol used for activating the inducible transgenic OSKM, as the pancreas, stomach and intestine tend to show better results when the doxycycline activator is administered via drinking water. In the case of AAV transduction, the liver and the spleen tend to be the most affected organs. This effect is also conditioned by the non-homogenous expression of OSKM in the different tissues, as some cell types of different organs express these factors more broadly and rapidly than others. These differential reprogramming rates could present a challenge in the application of this technology as a treatment for progeroid syndromes. A protocol that allows for efficient, safe and generalized levels of partial cell reprogramming in all organs must be developed in the future to allow for the full implementation of this technology. Even with all these challenges the results derived from *in vivo* partial reprogramming studies are relevant and promising. These results are also backed up by *in vitro* studies which show that reprogramming of human fibroblasts from HGPS patients and aged healthy individuals erases the epigenetic marks correlated with aging (Chen et al., 2017; Lee et al., 2020). All these results hint at the potential of cell reprogramming for the treatment of both accelerated and physiological aging, that potential however, does not mean that the road is exempted of challenges.

## 3 New approaches for cell reprogramming-based interventions in progeroid syndromes

Given the systemic nature of both physiological and accelerated aging, direct administration of reprogrammed cells is not feasible due to the local effects of this approach. Taking this into account, the only viable option left is the use of cell reprogramming *in vivo*. Most techniques for *in vivo* delivery of nucleic acids to cells are based on DNA. The use of DNA has several advantages, since it is more stable and has more potential delivery methods than RNA. However, both of these advantages can turn into disadvantages in this particular scenario. One of the most common ways to administer DNA *in vivo* is through the use of the aforementioned AAVs, which presents several challenges. AAVs are dose limited and, even though they do not cause the strong immune response associated with other viral vectors, they do cause a humoral response that can impede repeated administration (Senís et al., 2018; Wang et al., 2019). Furthermore, the association between the treatment with AAVs and the development of hepatocarcinomas has been described (Donsante et al., 2007; Wang et al., 2019). Another potential problem derives from the nature of the nucleic acids used in AAVs, as DNA tends to remain on cells for a quite long period of time, which could cause problems in achieving the short and repeated pulses of expression of OSKM needed for partial reprogramming. Many AAVs types also show a strong tropism to different organs, which could be problematic to achieve the aforementioned generalized partial reprogramming and also causing difficulties for the fine tuning of the dose. A viable alternative to DNA and AAVs would be the use of RNA, taking advantage of the different technologies for its *in vivo* delivery developed for mRNA based vaccines (Qiu et al., 2021). The use of mRNA encoding OSKM will grant transient yet strong expression of the four factors, while the low immunogenicity of lipid nanoparticles (LNPs) could allow for repeated administration, thus achieving both conditions needed for partial reprogramming. There are already developed formulations for the efficient delivery of mRNA *in vivo* using LNPs as well as alternatives to direct them to different organs (Algarni et al., 2022; Zhang et al., 2023). All these characteristics could make RNA the best alternative to achieve partial cell reprogramming *in vivo* in mouse models of progeroid syndromes without the risks of allogenic DNA insertion in their genome.

**FIGURE 2:**
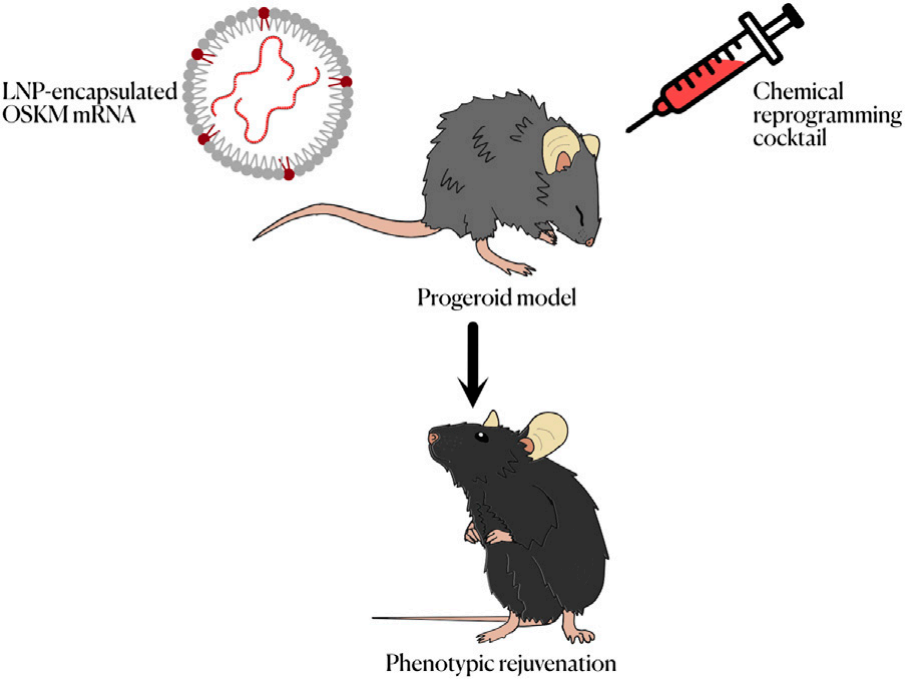
Summary of the different methods to achieve cell reprogramming *in vivo* for the treatment of progeroid syndromes.

The possibility of chemical reprogramming, which consists in the achievement of pluripotency from differentiated cells without the use of forced gene expression, should also be discussed. Instead of the expression of transcription factors, mixes of several small molecules acting as regulators of different cell processes, such as intracellular signaling cascades or epigenetic modification, are used to induce reprogramming. This approach has several advantages, including cost of manufacture of potential treatments, ease of administration and a greater dose-dependent effect. Somatic human cells have been returned to both a pluripotent and a partially reprogrammed state using different chemical cocktails (Guan et al., 2022; Schoenfeldt et al., 2022). This process was usually done using a four-stage protocol in which each step brought the cells up to a less differentiated stage. Recently this protocol, which lasted for 50 days, was shortened to a three stage protocol (Liuyang et al., 2023). This shorter protocol still achieves a great efficiency in the generation of iPSCs, yielding even better results that approaches based on the forced expression of transcription factors. These results are indeed promising, even though chemical reprogramming is based on the modification of the epigenome of the cells (Yang et al., 2023) and in the particular case of HGPS and other progeroid syndromes cell senescence is caused by DNA damage rather than by proliferative exhaustion and thus do not show an aged epigenome. This, however, is not necessarily a problem, as senescent cells derived from DNA damage are not the targets for reprogramming in a potential treatment. While more research on this topic is needed, chemical reprogramming constitutes a promising alternative to the use of OSKM for the potential treatment of progeroid syndromes. A combination with non cell reprogramming-based interventions aimed at counteracting the molecular alteration that drives accelerated aging, such as gene editing approaches targeting disease causing genes (Santiago-Fernández et al., 2019) could also be needed along with OSKM or chemical reprogramming approaches (Figure 2).

## 4 Conclusion

The potential of cell reprogramming to ameliorate the phenotype of murine models of accelerated aging has already been shown (Kim et al., 2023; Sahu et al., 2024). However, more efficient means of achieving transient and repeated expression of OSKM, or another way to induce partial cell reprogramming *in vivo*, needs to be developed before we can consider it for a potential treatment. In this regard, both new technologies for gene delivery as well as options for chemical reprogramming are promising alternatives. In order to be truly effective, however, this potential therapy would probably need to be accompanied by the treatment of the original cause of the disease. Without treating the root of the disease, the replenishment of stem cell populations could prove to be futile in the long term, as it was shown, for example, in the case of Parkinson’s disease (Tręda et al., 2024). Some advances in this regard have already been made, such as the use of AAVs containing gene editing CRISPR systems to prevent the expression of the prelamin A mRNA (Santiago-Fernández et al., 2019). A combined therapy targeting both the original cause and the derived damage would be the ideal scenario and a sensible direction in which research in this field should be heading. All in all, we can conclude that the use of cell reprogramming for the treatment of progeroid syndromes has a great potential but also a great deal of challenges to overcome. Much more research will be needed in the future to make it become a reality.

## Data Availability

The original contributions presented in the study are included in the article/supplementary material, further inquiries can be directed to the corresponding author.
